# Land use changes alter microbial functional gene diversity and its relationship with soil ecosystem multifunctionality in a subtropical estuary

**DOI:** 10.3389/fmicb.2025.1592901

**Published:** 2025-06-03

**Authors:** Zi-Kai Liu, Lige Zhang, Shengsheng Jin, Hanxia Yu, Ji-Zheng He, Ju-Pei Shen

**Affiliations:** ^1^Key Laboratory of Humid Subtropical Eco-Geographical Process of Ministry of Education, Fujian Normal University, Fuzhou, China; ^2^School of Geographical Sciences/School of Carbon Neutrality Future Technology, Fujian Normal University, Fuzhou, China

**Keywords:** coastal wetland, ecosystem multifunctionality, land use, microbial functional gene, salinity

## Abstract

Land use change in coastal wetlands is often associated with microbial diversity and function, which plays a crucial role in mediating soil ecosystem multifunctionality (EMF). However, the linkage between microbial functional genes and soil EMF under different land uses requires further investigation. This study investigated the relative abundance and community structure of microbial functional genes associated with carbon (C), nitrogen (N), phosphorus (P) and sulfur (S) cycling and their relationship with soil EMF across five different land uses (reed wetland, tidal flat, grassland, agricultural land and fallow land) in the Min River Estuary using high-throughput quantitative PCR technique. Results showed that microbial functional gene composition changed significantly across different land uses. Soil electrical conductivity (EC) ranged from 5.73 mS/cm (tidal flat) to 0.29 mS/cm (fallow land), driving significant shifts in microbial functional gene composition. Soil EMF exhibited a U-shaped trend across reed wetlands, tidal flats, grasslands, agricultural lands, and fallow lands, with the lowest in grasslands and peaking in fallow lands. Random forest analysis indicated that soil EC as the most influential environmental factor shaping microbial functional gene compositions, while functional gene richness directly correlated with EMF. Notably, soil EC modulates the relationship between microbial functional gene compositions and EMF. These findings enhance our understanding of soil EMF variations across different coastal land uses and underscore the need to integrate microbial functionality into coastal wetland management.

## 1 Introduction

Coastal wetlands are vital ecosystems that provide various ecological services, including carbon (C) sequestration, water purification, and habitats for diverse flora and fauna. Human activities have led to an estimated annual loss of approximately 1% of coastal habitats, including wetlands (Temmink et al., 2022). Changes in land use, such as converting wetlands for agricultural or aquaculture purposes, have a significant effect on ecosystem multifunctionality (EMF) (Tan et al., 2022), leading to land degradation (Haddad et al., 2015; Mao et al., 2018) and a decline in belowground biodiversity (Tang et al., 2021). Alterations in aboveground vegetation in wetlands can greatly influence belowground dynamics at both community and ecosystem levels (Abdu et al., 2016). Therefore, the consequences of land use change must be carefully considered in the management of coastal wetlands, particularly in the context of global change.

Land use changes significantly alter soil characteristics and biological attributes, particularly in coastal regions where ecosystems are highly sensitive to human interventions. For instance, the conversion of natural wetlands into agricultural fields is often associated with a marked decline in soil organic C and nitrogen (N) levels, as well as a substantial reduction in vegetation cover (Ding et al., 2013; Zhu X. et al., 2022). This degradation not only affects soil fertility but also disrupts ecological functions, such as C sequestration and nutrient cycling (Lal, 2004). Moreover, soil properties, including soil salinity (Hu et al., 2023b) and soil pH (O'Brien et al., 2019), often exhibit significant co-variation with total C and N contents, suggesting that these factors are interlinked through complex biogeochemical processes. Such changes in soil attributes can have cascading effects on microbial communities and plant diversity, further exacerbating ecosystem degradation (Schimel et al., 2007; Smith et al., 2015). Therefore, understanding the interactions between land use changes and soil properties is critical for designing sustainable land management strategies and mitigating the adverse impacts on coastal ecosystems.

Soil electrical conductivity (EC) is often used as a proxy for salinity, is widely acknowledged as a key edaphic factor shaping bacterial diversity (Siles and Margesin, 2016; O'Brien et al., 2019), and is one of the most variable parameters in terms of coastal land conversion. Microorganisms in coastal wetlands, such as reed wetlands, are accustomed to high-salinity habitats due to their natural habitat in the intertidal zone and must acclimate to lower salinity conditions if these areas are converted to cropland. Previous research has indicated that salinity decreases microbial α diversity in soils with lower salinity compared to seawater conditions (Hu et al., 2023a). Furthermore, numerous pieces of evidence has demonstrated that land conversion is often accompanied by change in microbial community diversity and composition (Wang Z. et al., 2022), and significant divergences in microbial community structure (Liang et al., 2024). However, the response of microbial functional groups to land use changes in estuary areas has not been well addressed.

Although previous studies have highlighted the importance of microbial community diversity, limited research has linked microbial functions to ecosystem processes (Tian et al., 2022). Alterations in the microbial community can influence biogeochemical cycling processes (Zhang et al., 2020; Bastida et al., 2021), such as nitrification (Liu et al., 2023), C fixation (Manoharan et al., 2017), sulfur (S) oxidation and reduction (Huang et al., 2023), and phosphorus (P) cycling (Hu et al., 2023b), consequently affecting soil EMF (Jing and He, 2021). For example, *nosZ* I and *nosZ* II have been used as biomarkers to quantify nitrous oxide (N_2_O) reducers (Jones et al., 2014; Xu et al., 2020). Meanwhile, genes *gcd* and *phoD* serve as biomarkers for microbial processes involved in P-solubilization and mineralization (Hu et al., 2023a). These functional genes are sensitive to land use changes and are associated with soil properties and ecosystem functions. For instance, low abundance of genes related to P cycling were detected in native grasslands than in croplands (Liu et al., 2018). The microbial community related to P cycling respond positively to moderate increases in salinity by enhancing microbial solubilization and mineralization of soil P, which, in turn, influences soil P availability and nutrient balance (Hu et al., 2023a). Moreover, changes in nutrient availability would affect soil enzyme activities and ecosystem functions (Delgado-Baquerizo et al., 2020). However, research addressing the response of a combination of the key biogeochemical processes to land use change remains limited. Additionally, land use change would simplify microbial diversity, leading to the reduction of soil EMF (Delgado-Baquerizo et al., 2016; Wang K. et al., 2022). Understanding the genetic basis of microbial functionality is essential for managing ecological processes and services, as microbial functional gene abundances are strongly associated with soil processes, thereby supporting soil functions and overall soil health (Jia et al., 2025). Land reclamation can enhance the activity and function of microbial groups (Gou et al., 2019). For example, soil microbial biomass increased in restored salt marshes in Netherlands, suggesting potential recovery of soil microbial function with appropriate management practices (Wu et al., 2015). However, other research has shown that artificial restoration significantly decreased soil EMF in alpine meadows (Wu et al., 2023). The inconsistent results may be attributed to multiple factors, such as land management practices and nutrient levels. Therefore, investigating the correlation between soil EMF and key microbial functional guilds is crucial for assessing the impacts of land use change on soil EMF in coastal wetland ecosystems.

The Min River Estuary in southeastern China is a typical intertidal zone along the eastern coast of China, that has experienced prolonged human interference. This region faces major threats, including sea-level rise, coastal erosion, human activities, and improper land management practices (Sun et al., 2015). We therefore hypothesized that land use changes in coastal wetlands will reduce microbial diversity and subsequently affect soil EMF. This study aimed to firstly investigate the impacts of different land uses on soil properties and microbial communities, and secondly to examine their associations with soil EMF in the Min River estuarine wetlands.

## 2 Material and methods

### 2.1 Study site

**Figure 1:**
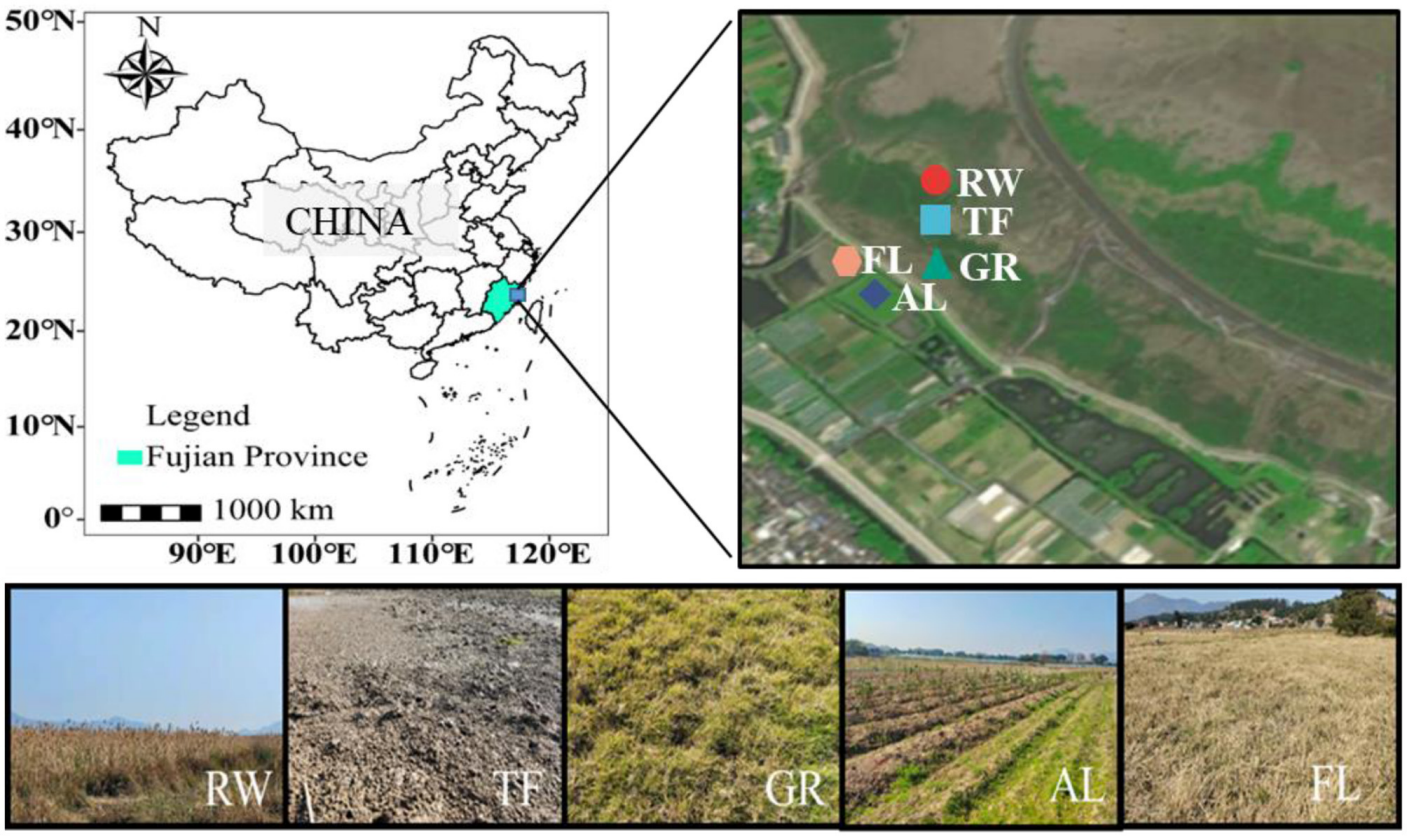
Sampling site location of Shanyutan tidal wetland and sampling point profile. Different colors and shapes represent different sampling areas. RW, reed wetland; TF, tidal flat; GR, grassland; AL, agricultural land; FL, fallow land.

This study was conducted in the Shanyutan tidal wetland of Min River Estuary (26° 1′-26°3′N, 119°36′-119°38′E), in Fujian province, China (Figure 1). This region is one of the most important tidal wetlands in southeast China (Tong et al., 2010; Zhang et al., 2015). The weather for this region is hot and rainy in the summer, while dry in the winter (Luo et al., 2016). The marsh soil type is dominated by saline soil (Li et al., 2020b). Soil descriptions and classifications followed the United States Department of Agriculture (USDA) (Li et al., 2020a). Based on the USDA classification system and a previous study, the soil textures in this area can be delineated as silt loam (> 70% silt and clay) (Luo et al., 2016). *Phragmites australis* is the dominant plant species in the reed wetland, a main component of the Shanyutan tidal wetland (Tong et al., 2010). The tidal flat is now a plain without vegetation cover. The reed wetland gradually transformed into grassland dominated by *C. compressus* due to the waterways changed (Gao et al., 2019). Agricultural land is located within the dam, where vegetables were grown at the time of sampling, and the land has been reclaimed for decades. Fallow land is also within the dam, with plants such as watercress, wormwood, and other shrubs, and has remained fallow for about a decade. To maintain wetland functionality, land managers have recently adopted fallowing as a reclamation strategy.

### 2.2 Soil sampling

The common land use types in the Shanyutan tidal wetland of the Min River Estuary include reed wetland (RW), tidal flat (TF), grassland (GR), agricultural land (AL), fallow land (FL), and aquaculture ponds (Zeng et al., 2008). In this study, five land use types including RW, TF, GR, AL, and FL were selected from this region in April 2021 (Shen et al., 2024) (Figure 1). RW here was considered original coastal wetland, while TF, GR and AL were the most recently impacted land use types. Land reclamation was implemented in FL. For the sampling process, five random soil cores, with 3.5 cm in diameter and 10 cm deep, were collected in each 5 m × 5 m plot. After removing plant material, fine roots, and gravel, soil samples from each plot were mixed and screened through a 2-mm mesh, resulting in a total of 15 soil samples. Each land use type included three plots with the distance over 10 m from each other. Soil samples were placed in the refrigerator and transported back to the laboratory for further analysis.

### 2.3 Soil properties determination

The determination of soil total dissolved organic carbon (DOC) and nitrogen (DON) and soil available N content including ${\text{NO}}_{3}^{-}$-N and ${\text{NH}}_{4}^{+}$-N were determined according to previous research (Hou et al., 2021; Deng et al., 2024; Shen et al., 2024). Soil salinity, indicated by electrical conductivity (EC) was measured using an EC meter, and soil pH was determined in a soil to water ratio of 1:2.5 with a pH meter (Bai et al., 2023). Soil total carbon (TC) and soil total nitrogen (TN) were analyzed using an element analyzer (VarioMAX, Elementar, Germany). Total phosphorus (TP) was determined using the molybdenum blue method at 880 nm with an automatic microplate reader (SPARK A-5082, Grodig, Austria) after extraction with the H_2_SO_4_-HClO_4_ fusion method. Available phosphorus (AP) was determined by M3 extract method and a continuous flow analyzer. Microbial biomass was determined using the chloroform fumigation method, and conversion factor was 0.45 and 0.54 for the calculation of microbial biomass C and biomass N, respectively (Li et al., 2021; Deng et al., 2024).

Four soil enzymes were included in this study: β-1, 4-glucosidase (βG), L-leucine aminopeptidase (LAP), acid phosphatase (ACP) and β-1, 4-n-acetylglucosaminidase (NAG), which are associated with sugar degradation, protein degradation, phosphorus mineralization, and chitin degradation, respectively. The detailed process for the determination of enzyme activities followed previous studies (Bell et al., 2013; Deng et al., 2024). Soil properties are listed in Supplementary Table 1.

### 2.4 High-throughput quantitative PCR analyses

Genomic DNA in soil samples was extracted from 0.5 g freezing samples using the FastDNA^®^ Spin Kit for Soil (MP Biomedicals, USA) and the obtained DNA concentration and quality were checked according to previous research (Deng et al., 2024).

High-throughput quantitative PCR was applied to detect the abundance of microbial functional genes in soil samples (WaferGen Biosystems, California, USA) (Li H. et al., 2020). The Quantitative Microbial Elemental Cycling (QMEC) method, a high-throughput quantitative PCR technique, was used to determine the relative abundance of microbial functional genes (Zheng et al., 2018), including 71 functional genes related to C, N, P, S metabolism. The primers information is listed in Supplementary Table 2. The quantitative PCR amplification cycles, amplification efficiency and threshold were carried out as previously (Zheng et al., 2018; Li H. et al., 2020), and the results were calculated based on previous study (Equation 1) (Zheng et al., 2018).


(1) $$\begin{array}{l} \text{Gene relative copy number }=\left(31-{\text{C}}_{\text{T}}\right)/\left(10/3\right) \end{array}$$

### 2.5 Soil ecosystem multifunctionality calculation

We calculated the EMF index using two standardized methods: Z-scores and 0-1 normalization. A total of 11 ecosystem functions, including nutrient parameters (${\text{NH}}_{4}^{+}$-N, ${\text{NO}}_{3}^{-}$-N, DOC, DON, AP, MBC and MBN) and enzyme activities (ACP, βG, NAG, LAP), were selected based on previous research (Deng et al., 2024), and other physicochemical properties, including TC, TN, TP, EC, and pH, were considered as environmental factors for downstream analysis. The average of the Z-scores for the measured variables was used as EMF for each sample for the first method (Jing et al., 2015). The second method involved standardizing each variable between 0 and 1 using the formula (Equation 2) (Delgado-Baquerizo et al., 2020; Jiao et al., 2022). The EMF index was obtained by averaging these standardized values.


(2) $$\begin{array}{l} \text{SV}=\left(\text{V}-\text{Vmin}\right)/\left(\text{Vmax}-\text{Vmin}\right) \end{array}$$

SV represents the standardized variable, and V, Vmin, and Vmax refer to the raw function, the min raw function, and the max raw function of each variable, respectively.

### 2.6 Statistical analyses

In this study, R environment (https://www.r-project.org/) was applied for statistical analysis. Functional gene richness, representing alpha diversity, was calculated based on the rarefied abundance tables, and non-metric multidimensional scaling (NMDS) analysis based on the relative abundance of each gene was performed to assess community structure using “vegan” package (Dixon, 2003). Random forest analysis was performed to identify the best predictors for functional gene community structure (NMDS1) and soil EMF using “randomForest” package in R (v.4.2.3). Mantel tests were conducted to reveal correlations between microbial functional gene community similarity and environmental factors. Correlations between microbial functional genes (C, N, P and S) composition and soil environmental factors were analyzed using the “ggcor” package. Regression analysis was performed using the “ggpmisc” package. Chord diagrams of functional genes were drawn using the “circlize” package. Structural equation modeling (SEM) was applied to evaluate the contribution of TN, EC, NMDS1 and functional gene richness to EMF. All the variables were included as independent observable variables and the modeling was computed using SPSS-Amos 28 software.

## 3 Results

### 3.1 Microbial functional gene diversity and their influencing factors

**Figure 2:**
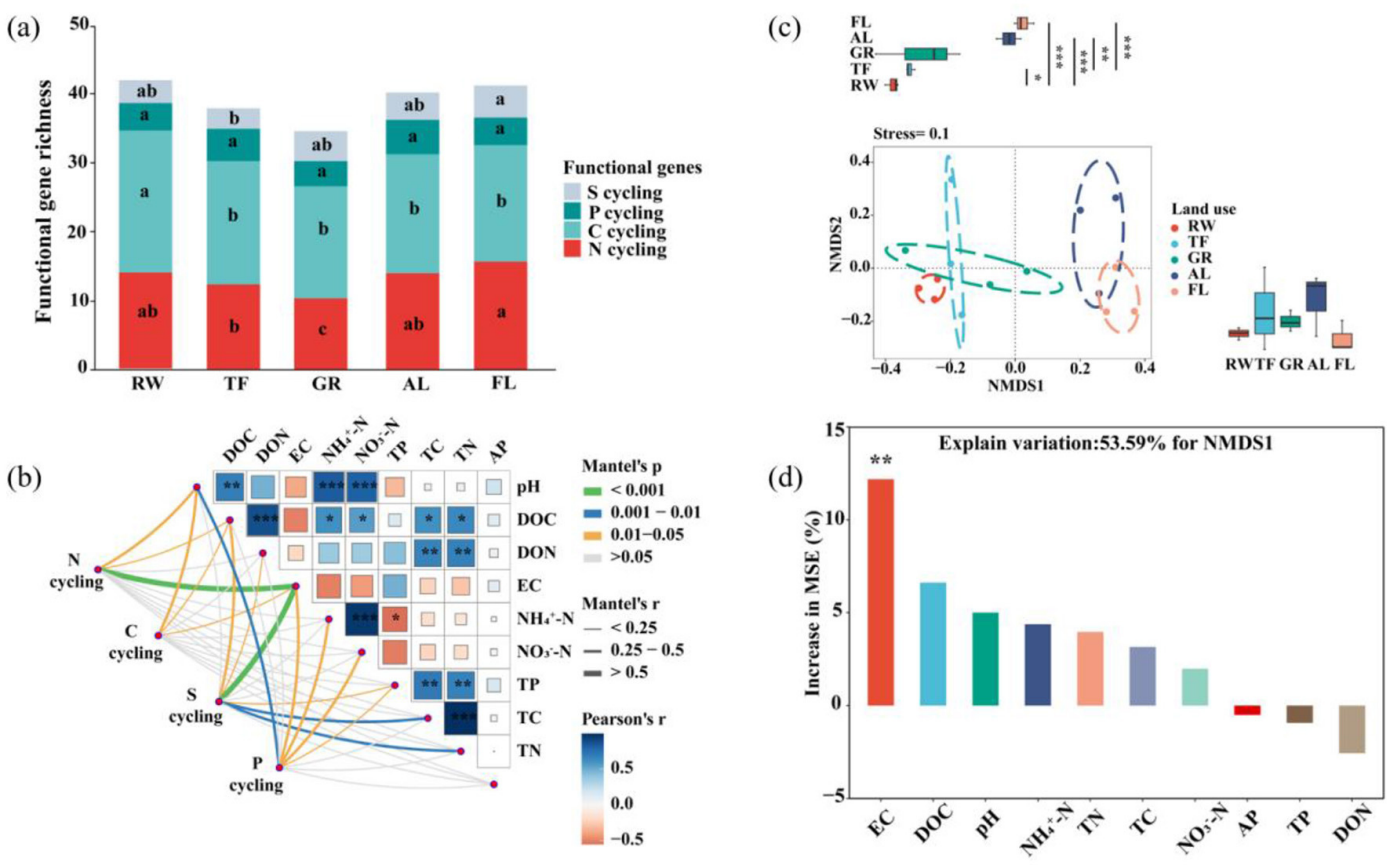
Distribution pattern and community structure of microbial functional gene Microbial functional genes richness **(a)**, mantel test showing the correlation between functional genes community composition and environmental factor **(b)**, non-metric multidimensional scaling (NMDS) analysis of functional gene community structure based on their abundance **(c)**, and Random forest analysis of factors affecting functional gene community structure **(d)**. Different letters indicate significant differences among different land use (*P* < 0.05). Pairwise comparisons of environmental factors are shown, with color gradient denoting Pearson's correlation coefficients. Edge width corresponds to the Mantel's r statistic for the correlations, and edge color denotes the statistical significance. Significance levels are denoted with ^*^P < 0.05, ^**^P < 0.01 and ^***^P < 0.001. pH, power of hydrogen; AP, Available phosphorus; DOC, dissolved carbon; DON, dissolved nitrogen; EC, electrical conductivity; ${\text{NH}}_{4}^{+}$-N, ammonium nitrogen; ${\text{NO}}_{3}^{-}$-N, nitrate nitrogen; TP, total phosphorus; TC, total carbon; TN, total nitrogen. Significance levels are denoted with ^*^P < 0.05, ^**^P < 0.01, ^***^P < 0.001. Different colors represent different land use and functional genes. RW, reed wetland; TF, tidal flat; GR, grassland; AL, agricultural land; FL, fallow land.

Soil properties exhibited significant variation across different land uses (Supplementary Table 1). Generally, soil pH, EC, and nutrient parameters all showed a U-shaped pattern from RW, TF, GR, AL to FL. A total of 55 functional genes related to key nutrient element cycling were detected. Among these, the largest number of genes was found in C cycling around 27, and S cycling genes were the fewest around 5 (Figure 2a). The richness of microbial functional genes decreased from RW to TF and GR, then increased in AL and FL. The highest richness was in RW (43), and the lowest was in GR (34) (Figure 2a). Pearson analysis indicated significant correlations between soil ${\text{NH}}_{4}^{+}$-N, DOC and ${\text{NO}}_{3}^{-}$-N with soil pH (*P* < 0.05) (Figure 2b). Soil DOC and DON were noteworthy correlated with TC and TN (*P* < 0.05). Notably, DOC was significantly correlated with soil ${\text{NH}}_{4}^{+}$-N and ${\text{NO}}_{3}^{-}$-N, while TP showed a significantly negative correlation with ${\text{NH}}_{4}^{+}$-N (*P* < 0.05).

Mantel test revealed that soil EC was the strongest driver of the relative abundance of microbial functional genes. Additionally, soil pH was significantly correlated with the relative abundance of those microbial genes related to C, N and P cycling, and soil DOC with those involved in C, N and S cycling (Figure 2b). NMDS analysis showed a distinct distribution pattern among land uses, clustering into three groups: RW and TF, GR, AL and FL. The first two groups were significantly separated from the third group along the x-axis (Figure 2c). Random forest analysis identified that soil EC was the most significant contributor to the community structure of microbial functional genes (NMDS1) (Figure 2d).

### 3.2 Top 10 functional gene differences and correlation analysis with soil properties

**Figure 3:**
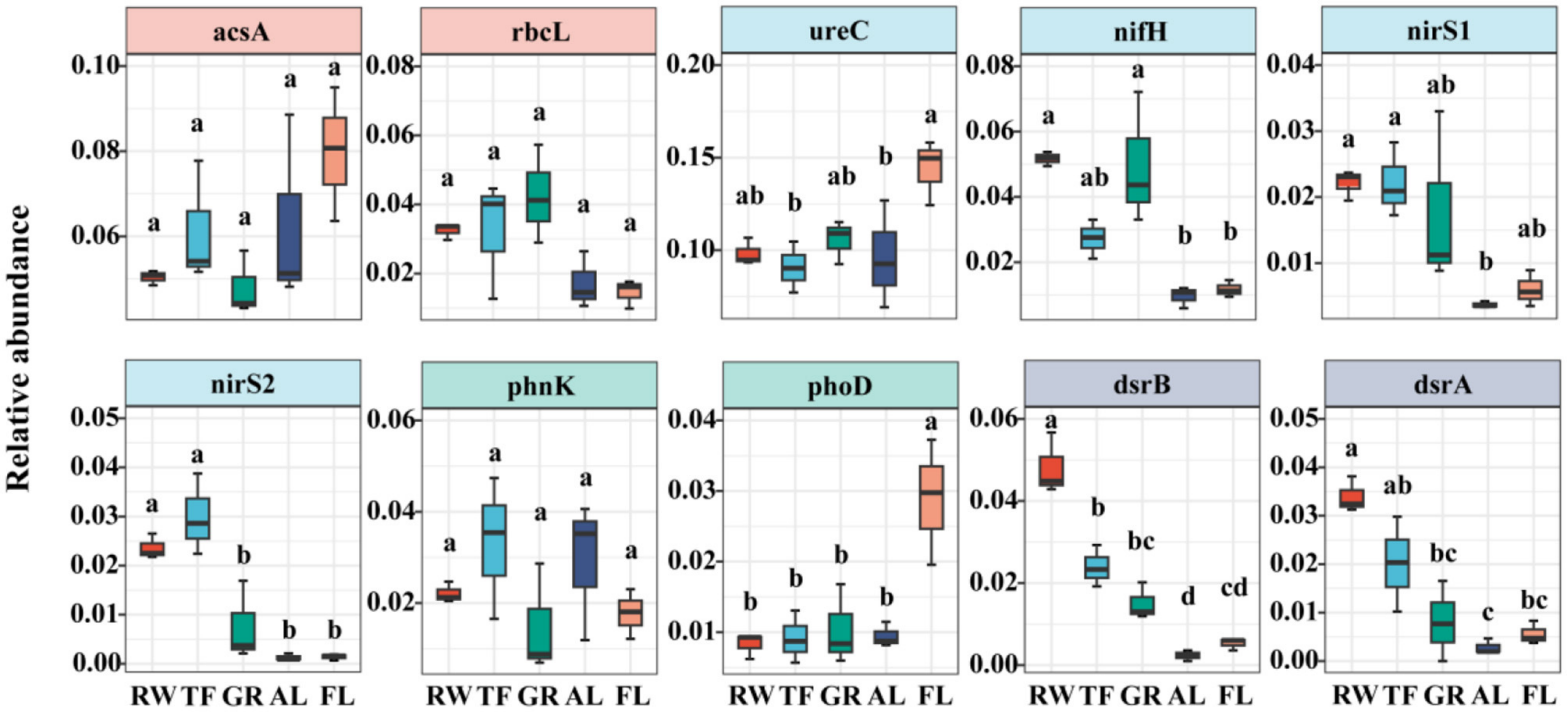
Relative abundance of top 10 microbial functional genes in different land use. Different lowercase letters above the bar indicate significant differences among different land use (*P* < 0.05). Different colors represent different land uses. RW, reed wetland; TF, tidal flat; GR, grassland; AL, agricultural land; FL, fallow land.

The top 10 abundant microbial functional genes were shown in this study (Supplementary Figure 1), and *ureC* gene had the peak relative abundance in all the samples, followed by *arcsA, nifH, rbcL, phnK, dsrB, nirS1, dsrA, phoD*, and *nirS2* genes. ANOVA analysis of the relative abundance of top 10 genes showed varied patterns across land uses (Figure 3). For example, genes *nifH, dsrB, nirS1, dsrA*, and *nirS2* showed a decreasing trend from RW to FL, whereas, the relative abundances of *ureC* and *phoD* genes showed an opposite trend, with the highest found in AL. No significant differences in the relative abundances of *acsA, rbcL* and *phnK* genes were found across different land uses.

**Figure 4:**
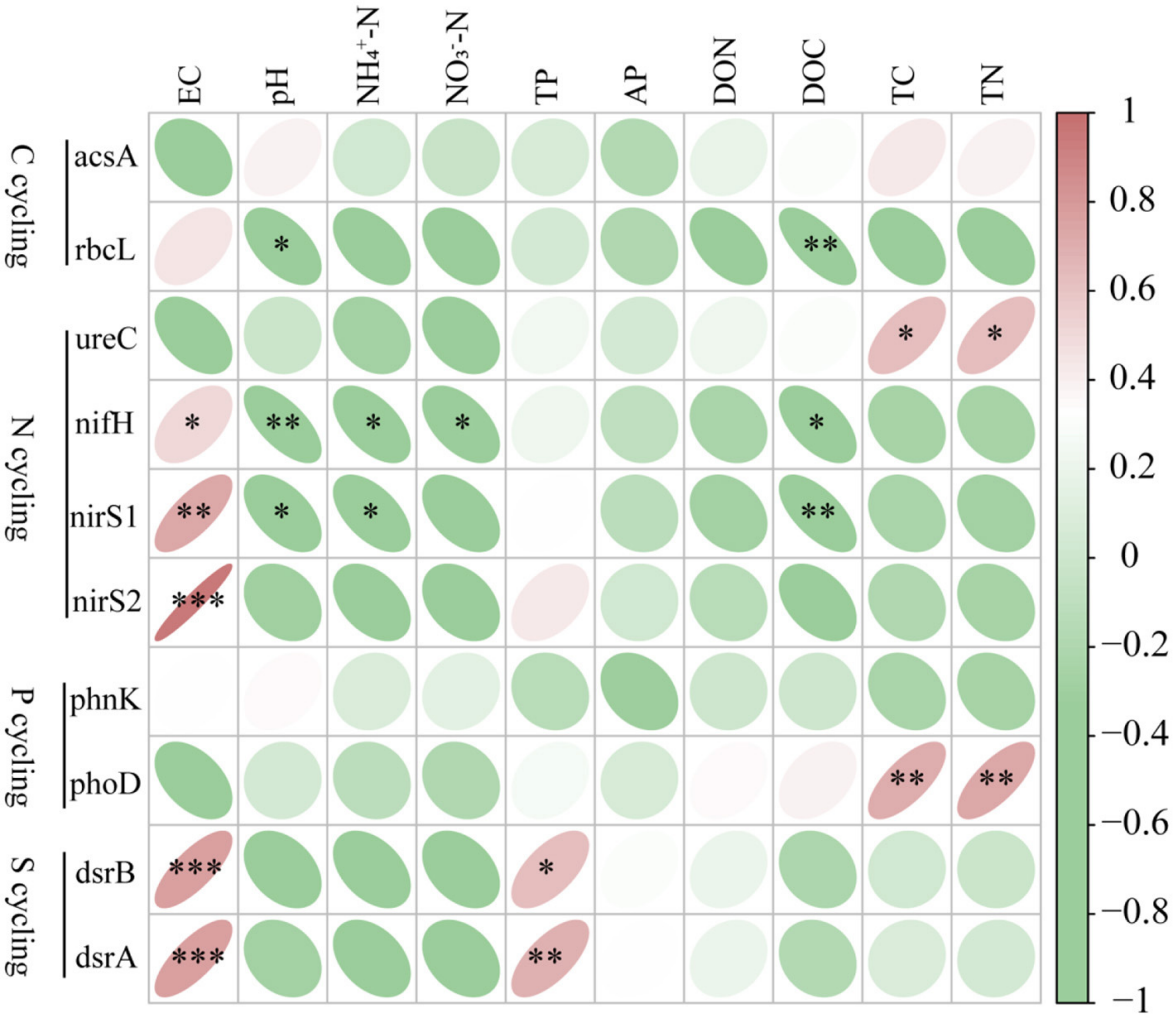
Correlation analysis of top 10 functional genes abundance and environmental factors. Significance levels are denoted with *P < 0.05, **P < 0.01 and ***P < 0.001. pH, power of hydrogen; AP, Available phosphorus; DOC, dissolved carbon; DON, dissolved nitrogen; EC, electrical conductivity; ${\text{NH}}_{4}^{+}$-N, ammonium nitrogen; ${\text{NO}}_{3}^{-}$-N, nitrate nitrogen; TP, total phosphorus; TC, total carbon; TN, total nitrogen. Red means positive correlation, green means negative correlation.

Significant positive correlations between microbial functional genes abundance and environmental variables were observed (Figure 4). Significant positive correlations were detected between microbial functional genes and soil properties, such as *nifH, dsrB, nirS1, dsrA*, and *nirS2* with EC, *dsrB* and *dsrA* with TP, and *ureC and phoD* with TC and TN (Figure 4). Conversely, significant negative correlations were found, between *nifH* and pH, ${\text{NH}}_{4}^{+}$-N, NO3^−^-N, DOC, between *rbcL* and pH, DOC; and between *nirS1* and pH, ${\text{NH}}_{4}^{+}$-N, DOC. These results suggest that environmental variables significantly influence the relative abundance of microbial genes.

**Figure 5:**
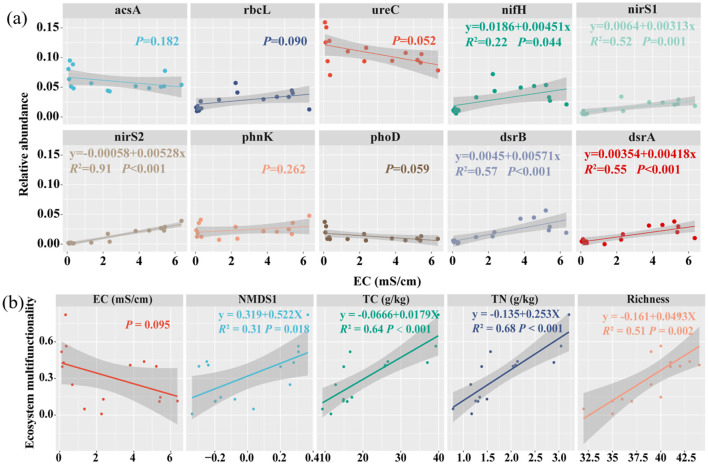
Regression analysis of functional genes and EMF. Soil EC and top 10 functional genes **(a)** and soil EMF, EC, NMDS1and richness, TC and TN **(b)**. The shady region shows the regression model's 95 % confidence interval. Richness: microbial functional genes richness, NMDS1: the first axis of non-metric multidimensional scaling (NMDS) Different colors represent different genes and variables.

Regression analysis indicated that soil EC exhibited a negative correlation with functional gene NMDS1 (*P* < 0.05) (Supplementary Figure 2). Regression analysis further demonstrated that soil EC plays a major role in the distribution of the top 10 functional genes, with significant (*P* < 0.05) *R*^2^ value: 0.83 for *nirS2*, 0.62 for *nirS1* and *dsrB*, 0.57 for *dsrA*, 0.40 for *nifH*, 0.29 for *rbcL*, 0.27 for *phoD*, and 0.23 for *ureC* (Figure 5).

### 3.3 Soil EMF and driving factors across different land use

**Figure 6:**
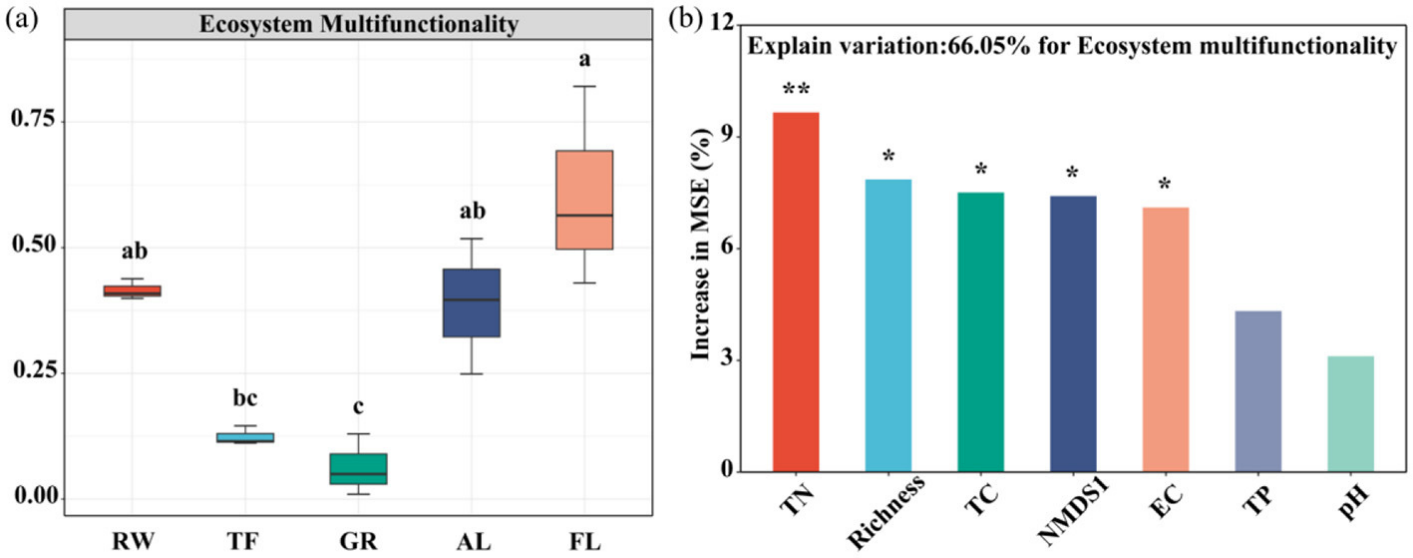
Soil EMF in different land uses **(a)** and random forest analysis of factors affecting EMF **(b)**. Different letters indicate significant differences among different land use (*P* < 0.05). RW, reed wetland; TF, tidal flat; GR, grassland; AL, agricultural land; FL, fallow land. Significance levels are denoted with *P < 0.05, **P < 0.01. pH, power of hydrogen; EC, electrical conductivity; TP, total phosphorus; TC, total carbon; TN, total nitrogen; Richness, microbial functional genes richness; NMDS1, microbial functional genes community structure. Different colors represent different variables.

Soil EMF based on both the 0-1 normalization and Z-scores methods showed a U-shaped trend across the land use types, from RW, FL, including TF, GR, to AL (Figure 6a, Supplementary Figure 3). The lowest EMF was recorded in GR, while no significant differences were observed between RW, AL and FL. This consistency supports the reliability of our EMF values. Given its widespread use in the literature, we ultimately chose the 0-1 normalization method in the downstream analysis.

Random forest analysis indicated that the relative abundance of the *dsrB, nifH, rbcL, nirS2*, and *dsrA* genes were the major microbial factors contributing to soil EMF (Supplementary Figure 4). Furthermore, TN, microbial functional gene richness, TC, NMDS1 and EC were identified as major factors influencing soil EMF (Figure 6b). Regression analysis indicated that microbial community structure (NMDS1), TC, TN, and functional gene richness all have noteworthy correlations with soil EMF (*P* < 0.05) (Figure 5).

**Figure 7:**
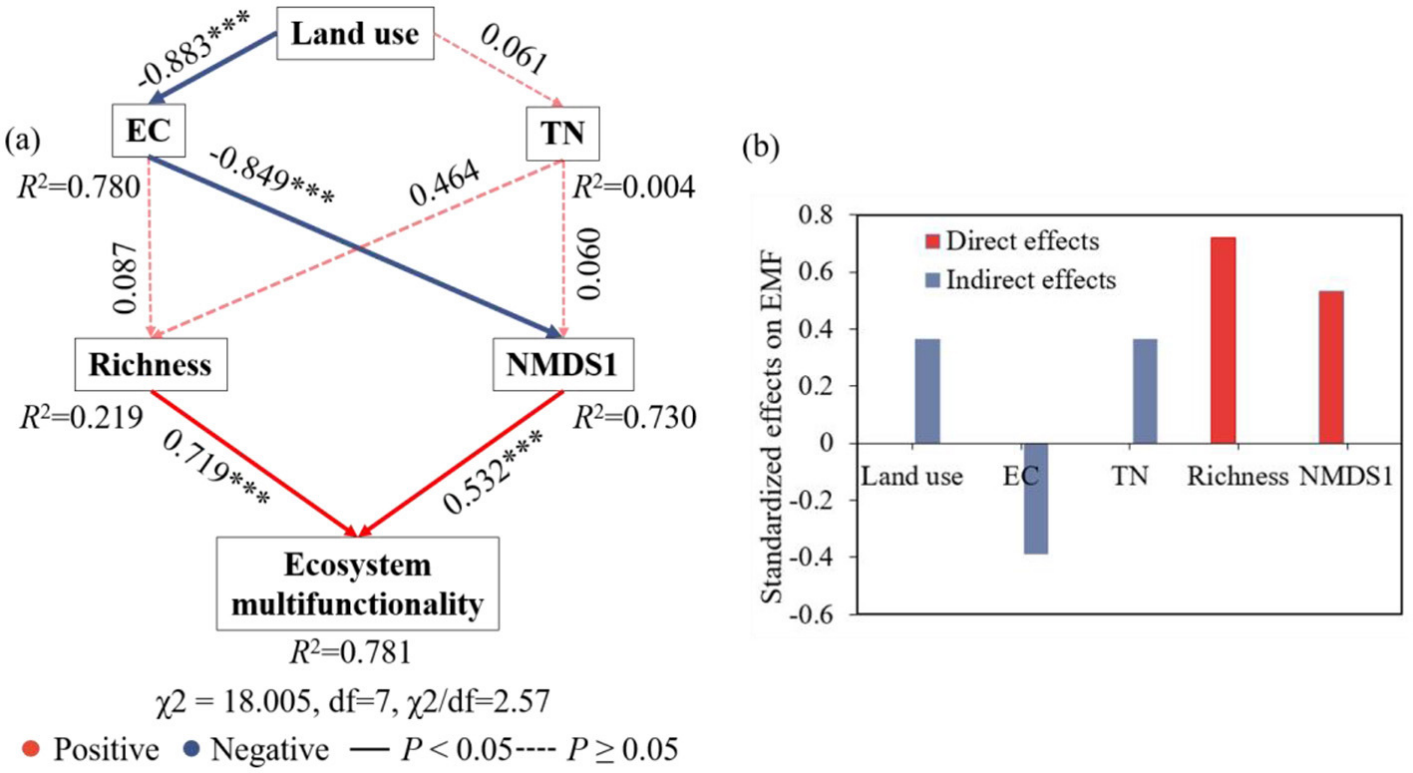
Structural equation modeling (SEM) showing the TN, EC, NMDS1, microbial functional gene richness and EMF **(a)**. EC, electrical conductivity; TN, total nitrogen. Richness, microbial functional genes richness; NMDS1, the first axis of non-metric multidimensional scaling (NMDS). Adjacent number in the same direction as the arrow represents path coefficients. Solid and dotted lines represent significant (*P < 0.05) and non-significant relationships, respectively, with red represents positive correlation and blue gray represents negative correlation, and significance levels denoted as *P < 0.05, **P < 0.01, and ***P < 0.001. The standardized effects of multiple factors on the soil EMF **(b)**. Different colors represent different effects.

SEM approach was employed to further investigate the direct and indirect factors contributing to soil EMF. Results indicated that soil EC indirectly affects EMF through microbial functional gene community structure (NMDS1) (Figure 7a). The model also showed that microbial functional gene richness had a positive impact on EMF. Additionally, it was found that soil EC and TN had no direct significant impact on the richness and NMDS1 of microbial functional genes. By calculating the effects of all variables on SEM, we found that the gene richness had the strongest impact on EMF, followed by microbial community structure (NMDS1) (Figure 7b).

## 4 Discussion

### 4.1 Effect of land use on soil properties and microbial functional genes

Coastal estuarine wetlands play a vital role in regulating biogeochemical cycles between land and sea (Wang K. et al., 2022). In this study, we found significant variations in soil properties across different land uses, which is consistent with the findings of previous studies (Ding et al., 2013; Zhu X. et al., 2022). This variation is partly attributed to land use and reclamation duration (Sun et al., 2011), which were well addressed in our previous study (Liu et al., 2022). Estuarine wetlands are particularly vulnerable to subtle environmental changes due to their unique geographical positions. Land use changes can affect soil physicochemical properties, biogeochemical processes, and consequently influence ecosystem function (Feng et al., 2022). Soil salinity is the most influential in coastal regions (Lorrain-Soligon et al., 2023). Our results further corroborate this conclusion by revealing marked salinity gradients from intertidal to nearshore zones (Zhang et al., 2020).

Land use changes alter soil physicochemical properties and microbial habitat conditions, leading to distinct characteristics in microbial community composition and microbe-mediated nutrient cycles, thereby shaping functional gene profiles (Dong et al., 2020; Mohapatra et al., 2021). Our findings indicated that significant difference in the community structure of microbial functional genes between intertidal and offshore zones, corroborating previous research by Zhu Y. et al. (2022). They observed notable distinctions in the bacterial abundance and community structure across various land reclamation periods in the Yellow River Delta (Zhu Y. et al., 2022). Furthermore, the response of microbial functional genes varied when natural land converted to agricultural habitats. For example, the transition from steppe to farmland diversified the spatial distribution of functional genes, resulting in a higher abundance of specific functional genes (Liu et al., 2023). However, a recent study showed that the transition from natural ecosystems to agricultural soils would result in the classification and functional homogenization of soil bacteria (Peng et al., 2024). In this study, we found *nifH, dsrB, nirS1, dsrA*, and *nirS2* genes showed a decreasing trend along RW, TF, GR, AL and FL (Figure 3). This trend is largely attributed to the microhabitat-specific characteristics of microorganisms. For example, *nirS1* and *nirS2* genes encoding for cytochrome cd1-containing reductase (NirS), primarily drive denitrification via denitrifying bacteria, which generally prefer anaerobic systems, such as river sediments (Li et al., 2014), sediment subsurface (Smith et al., 2007). The genes *dsrA* and *dsrB* encode dissimilatory sulfite reductase (DsrAB), which catalyzes the reduction of sulfite to sulfide during sulfate anaerobic respiration (Pelikan et al., 2016). RW and TF, which are located in the intertidal zones with limited O_2_ availability, exhibited higher numbers and relative abundances of *dsrB, nirS1, dsrA*, and *nirS2*. The *nifH* gene involved in N fixation was often affected by the availability of N and aboveground vegetation (Chen et al., 2019).

Salinity stress could inhibit plant growth and photosynthetic rate. Previous research has established that salinity as a critical soil characteristic affecting microbial communities and associated biogeochemical cycles, especially in estuary (Sheng et al., 2015). Salinity induces osmotic stress, altering microbial metabolism and the abundance of functional genes (e.g., *nosZ, nirK, pccA*). It also affects nutrient availability and microbial competition, reshaping community composition and ecosystem functions (Li et al., 2022, 2024). In subtropical coastal wetlands, salinity is the main factor affecting N cycling genes like *nosZ I* and *nosZ II* (Lin et al., 2023). In this study, soil EC was the dominant factor driving functional gene community structure and was positive correlations with soil microbial functional genes, including *nifH, dsrB, nirS1, dsrA*, and *nirS2*. Long-term fluctuations in seawater alter the growth environment for vegetation in intertidal and offshore zones (Costa et al., 2003). The continuous accumulation of salts by plant roots and litter leads to the flocculation and buildup of humic substances under high salinity conditions in coastal wetlands (Kida et al., 2017; Li et al., 2022; Lin et al., 2023). Intertidal vegetation significantly influences the abundance of microbial functional genes through root exudates and plant litter (Chaudhary et al., 2018). In this study, land use conversion significantly affected soil pH, which is a crucial factor affecting microbial diversity and composition (Lauber et al., 2009). Nevertheless, the influence of soil pH on microbial functional gene community was less significant than that of soil EC, which is consistent with findings in saline soils (Mutlu et al., 2008). Soil salinity strongly drives microbial community structure and function (Zhang et al., 2020), particularly in coastal estuaries (O'Brien et al., 2019). Alterations in the composition and diversity of microbial functional genes in saline soils in coastal zone provide critical biological indicators for land use transitions.

### 4.2 Role of soil EMF in coastal wetland

Our findings indicated that soil EMF varied significantly across five land uses. Fallow land had the highest soil EMF among the five land uses. Soil EMF was positively correlated with microbial functional gene richness, and both decreased initially from reed wetlands to fallow land. Land use changes play a crucial role in regulating EMF by driving key biogeochemical cycles. In saline soils, reductions in microbial C, N, and P metabolism alter nutrient availability and reshape community composition, ultimately influencing EMF (Hu et al., 2023a; Liu et al., 2023). Consequently, microbial functional genes directly regulate key soil processes, such as nutrient transformation and organic matter decomposition, which in turn support overall soil EMF (Li et al., 2022, 2024; Luo et al., 2025). Biodiversity has been considered as a major contributor to EMF in various environments (Wagg et al., 2014), including grassland (Guo et al., 2021; Ding et al., 2025) and agricultural systems (Jiao et al., 2021; Deng et al., 2024). In this study, soil EMF was strongly associated with the richness of microbial functional genes as confirmed by SEM analysis. Previous studies have demonstrated that soil food web structure (e.g., trophic interactions among bacteria, fungi, and nematodes) and microbial community composition play crucial roles in regulating EMF (Zhu et al., 2024; Ding et al., 2025). While functional gene richness more directly reflects microbial metabolic potential, linking biogeochemical processes such as C and N cycling. This suggests that interrogating biodiversity at multiple biological scales, i.e., from genes to communities or food webs, provides a holistic view of how biodiversity sustains ecosystem services. Additionally, we also found that soil TC and TN all showed strong correlations with soil EMF, but not with soil EC. Soil salinity is mainly associated with parent materials and local habitats, such as coastal wetlands (Zhang et al., 2024). However, the effects of salinity on microbial activity and function are generally mitigated when coastal wetlands are converted to other upland types, such as agricultural land. Both microbial functional gene richness and community structure explained more variation in soil EMF than soil EC, although soil EC is the best predictor for microbial gene community. This is likely due to other factors combined with microbial characteristics driving soil EMF, such as TC and TN. Previous research demonstrated that nitrogen application can improve nutrients characteristics and microbial metabolic activities in coastal agro-ecosystems by creating more suitable bacterial microhabitats (Yao et al., 2021). This aligns with our finding that higher soil EMF were recorded in AL and FL, which received fertilizers and litter inputs, respectively. In this study, fertilization benefits agricultural land (AL) by enhancing nutrients, while fallow land (FL) maintains high C and N levels due to minimal disturbance (Supplementary Table 1). Our results extend the understanding of the links between soil microbial communities and EMF and further clarify their environmental implications.

Soil EMF provides a comprehensive assessment of soil functions. Previous studies of EMF are mostly related to plant productivity, vegetation biomass, and nutrient cycling (Delgado-Baquerizo et al., 2016, 2020). Natural ecosystems exhibit highly complex taxonomic diversity and environmental heterogeneity, making it challenging to understand the factors influencing subsurface electromagnetic fields (Jiao et al., 2021). Soil biodiversity, especially microbial diversity, is an important part for maintaining ecosystem function (Delgado-Baquerizo et al., 2016). In addition, previous research found that soil EMF significantly increased during ecosystem restoration (Tian et al., 2022). During the restoration process, increased plant litter and soil microbial activity enhance litter decomposition and nutrients transformation (Zhang et al., 2022). Higher biodiversity and diverse ecosystem functions ultimately promote ecosystem assembly and significantly enhance restoration outcomes (Guo et al., 2021). Future research should combine biodiversity and EMF together to better assess land conservation and ecosystem restoration efforts.

## 5 Conclusion

This study elucidates the interplay between land use change, microbial functional communities, and soil EMF in subtropical coastal wetlands. Soil EC emerged as the dominant factor structuring microbial functional communities, yet EMF was more strongly influenced by microbial gene richness and nutrient dynamics (i.e., TC, TN). Fallow land characterized with low disturbance exhibited the highest EMF, highlighting the ecological benefits of land restoration. Conversely, the conversion of wetlands to grassland reduced EMF. Future research should integrate multi-trophic interactions and long-term monitoring to refine land management strategies. These findings emphasize the critical role of soil microbial diversity and nutrient cycling in maintaining ecosystem services and provide practical insights for minimizing anthropogenic impacts while promoting sustainable wetland management.

## Data Availability

The original contributions presented in the study are included in the article/Supplementary material, further inquiries can be directed to the corresponding author.

## References

[B1] Abdu N. Abdullahi A. A. Abdulkadir A. (2016). Heavy metals and soil microbes. Environ. Chem. Lett. 15, 65–84. 10.1007/s10311-016-0587-x

[B2] Bai Y. Wei H. Ming A. Shu W. Shen W. (2023). Tree species mixing begets admixture of soil microbial communities: variations along bulk soil, rhizosphere soil and root tissue. Geoderma 438:116638. 10.1016/j.geoderma.2023.116638

[B3] Bastida F. Eldridge D. J. García C. Png G. K. Bardgett R. D. Delgado-Baquerizo M.. (2021). Soil microbial diversity-biomass relationships are driven by soil carbon content across global biomes. ISME J. 15, 2081–2091. 10.1038/s41396-021-00906-0 33564112 PMC8245509

[B4] Bell C. W. Fricks B. E. Rocca J. D. Steinweg J. M. McMahon S. K. Wallenstein M. D.. (2013). High-throughput fluorometric measurement of potential soil extracellular enzyme activities. J. Visualized Exp. 81:e50961. 10.3791/50961-v 24299913 PMC3991303

[B5] Chaudhary D. R. Kim J. Kang H. (2018). Influences of different halophyte vegetation on soil microbial community at temperate salt marsh. Microb. Ecol. 75, 729–738. 10.1007/s00248-017-1083-y 28986657

[B6] Chen J. Shen W. Xu H. Li Y. Luo T. (2019). The composition of nitrogen-fixing microorganisms correlates with soil nitrogen content during reforestation: a comparison between legume and non-legume plantations. Front. Microbiol. 10:508. 10.3389/fmicb.2019.00508 30930882 PMC6427063

[B7] Costa C. S. B. Marangoni J. C. Azevedo A. M. G. (2003). Plant zonation in irregularly flooded salt marshes: relative importance of stress tolerance and biological interactions. J. Ecol. 91, 951–965. 10.1046/j.1365-2745.2003.00821.x

[B8] Delgado-Baquerizo M. Maestre F. T. Reich P. B. Jeffries T. C. Gaitan J. J. Encinar D.. (2016). Microbial diversity drives multifunctionality in terrestrial ecosystems. Nat. Commun. 7:10541. 10.1038/ncomms10541 26817514 PMC4738359

[B9] Delgado-Baquerizo M. Reich P. B. Trivedi C. Eldridge D. J. Abades S. Alfaro F. D.. (2020). Multiple elements of soil biodiversity drive ecosystem functions across biomes. Nat. Ecol. Evol. 4, 210–220. 10.1038/s41559-019-1084-y 32015427

[B10] Deng H. Ma X. Liu Z. Hu H. Di H. J. Liu Y.. (2024). Soil ecosystem multifunctionality is strongly linked with crop yield after four decades chemical fertilization in black soil. Agric. Ecosyst. Environ. 368:109007. 10.1016/j.agee.2024.109007

[B11] Ding C. Liu Y. Hernández M. Sun H. Jiao S. Pan H.. (2025). Coupling soil bacterial and fungal community traits to multifunctionality in grassland ecosystem. Agric. Ecosyst. Environ. 388:109648. 10.1016/j.agee.2025.109648

[B12] Ding F. Hu Y. L. Li L. J. Li A. Shi S. W. Lian P. Y.. (2013). Changes in soil organic carbon and total nitrogen stocks after conversion of meadow to cropland in Northeast China. Plant Soil 373, 659–672. 10.1007/s11104-013-1827-5

[B13] Dixon P. (2003). VEGAN, a package of R functions for community ecology. J. Veg. Sci. 14, 927–930. 10.1111/j.1654-1103.2003.tb02228.x

[B14] Dong S. Li Y. Ganjurjav H. Gao Q. Gao X. Zhang J.. (2020). Grazing promoted soil microbial functional genes for regulating C and N cycling in alpine meadow of the Qinghai-Tibetan Plateau. Agric. Ecosyst. Environ. 303:107111. 10.1016/j.agee.2020.107111

[B15] Feng Z. Wang L. Peng Q. Li J. Liang T. (2022). Effect of environmental factors on soil properties under different land use types in a typical basin of the North China Plain. J. Cleaner Prod. 344:131084. 10.1016/j.jclepro.2022.131084

[B16] Gao H. Zhai S. Sun Z. Liu J. Tong C. (2019). Differences in biomass and silica content in typical plant communities with ecotones in the Min River estuary of southeast China. PeerJ 7:e7218. 10.7717/peerj.7218 31367481 PMC6657677

[B17] Gou X. Hu J. Chen Y. Wei X. Du Z. Zhou Q.. (2019). The effect of artificial vegetation recovery on the soil nutrients and enzyme activities in subhumid desert land on the southeast Qinghai-Tibetan Plateau, China. Ecol. Eng. 139:105528. 10.1016/j.ecoleng.2019.06.023

[B18] Guo Y. Xu T. Cheng J. Wei G. Lin Y. (2021). Above- and belowground biodiversity drives soil multifunctionality along a long-term grassland restoration chronosequence. Sci. Total Environ. 772:145010. 10.1016/j.scitotenv.2021.145010 33578173

[B19] Haddad N. M. Brudvig L. A. Clobert J. Davies K. F. Gonzalez A. Holt R. D.. (2015). Habitat fragmentation and its lasting impact on Earth's ecosystems. Sci. Adv. 1:e1500052. 10.1126/sciadv.1500052 26601154 PMC4643828

[B20] Hou Q. Zuo T. Wang J. Huang S. Wang X. Yao L.. (2021). Responses of nitrification and bacterial community in three size aggregates of paddy soil to both of initial fertility and biochar addition. Appl. Soil Ecol. 166:104004. 10.1016/j.apsoil.2021.104004

[B21] Hu M. Le Y. Sardans J. Yan R. Zhong Y. Sun D.. (2023a). Moderate salinity improves the availability of soil P by regulating P-cycling microbial communities in coastal wetlands. Glob. Change Biol. 29, 276–288. 10.1111/gcb.16465 36181699

[B22] Hu M. Yan R. Wu H. Ni R. Zhang D. Zou S.. (2023b). Linking soil phosphorus availability and phosphatase functional genes to coastal marsh erosion: implications for nutrient cycling and wetland restoration. Sci. Total Environ. 898:165559. 10.1016/j.scitotenv.2023.165559 37454858

[B23] Huang J. Liu X. Liu J. Zhang Z. Zhang W. Qi Y.. (2023). Changes of soil bacterial community, network structure, and carbon, nitrogen and sulfur functional genes under different land use types. CATENA 231:107385. 10.1016/j.catena.2023.107385

[B24] Jia J. Goede d. e. Li R. Zhang Y. Wang J. Zhang G. J.. (2025). Unlocking soil health: are microbial functional genes effective indicators? Soil Biol. Biochem. 204:109768. 10.1016/j.soilbio.2025.109768

[B25] Jiao S. Lu Y. Wei G. (2021). Soil multitrophic network complexity enhances the link between biodiversity and multifunctionality in agricultural systems. Glob. Change Biol. 28, 140–153. 10.1111/gcb.15917 34610173

[B26] Jiao S. Qi J. Jin C. Liu Y. Wang Y. Pan H.. (2022). Core phylotypes enhance the resistance of soil microbiome to environmental changes to maintain multifunctionality in agricultural ecosystems. Glob. Change Biol. 28, 6653–6664. 10.1111/gcb.16387 36002985

[B27] Jing X. He J-. S. (2021). Relationship between biodiversity, ecosystem multifunctionality and multiserviceability: literature overview and research advances. Chin. J. Plant Ecol. 45, 1094–1111. 10.17521/cjpe.2020.0154

[B28] Jing X. Sanders N. J. Shi Y. Chu H. Classen A. T. Zhao K.. (2015). The links between ecosystem multifunctionality and above- and belowground biodiversity are mediated by climate. Nat. Commun. 6:8159. 10.1038/ncomms9159 26328906 PMC4569729

[B29] Jones C. M. Spor A. Brennan F. P. Breuil M-. C. Bru D. Lemanceau P.. (2014). Recently identified microbial guild mediates soil N2O sink capacity. Nat. Clim. Change 4, 801–805. 10.1038/nclimate2301

[B30] Kida M. Tomotsune M. Iimura Y. Kinjo K. Ohtsuka T. Fujitake N.. (2017). High salinity leads to accumulation of soil organic carbon in mangrove soil. Chemosphere 177, 51–55. 10.1016/j.chemosphere.2017.02.074 28282623

[B31] Lal R. (2004). Soil carbon sequestration impacts on global climate change and food security. Science 304, 1623–1627. 10.1126/science.1097396 15192216

[B32] Lauber C. L. Hamady M. Knight R. Fierer N. (2009). Pyrosequencing-based assessment of soil pH as a predictor of soil bacterial community structure at the continental scale. Appl. Environ. Microbiol. 75, 5111–5120. 10.1128/AEM.00335-09 19502440 PMC2725504

[B33] Li H. Bi Q. Yang K. Lasson S. B. Zheng B. Cui L.. (2020). High starter phosphorus fertilization facilitates soil phosphorus turnover by promoting microbial functional interaction in an arable soil. J. Environ. Sci. 94, 179–185. 10.1016/j.jes.2020.03.040 32563482

[B34] Li J. Cui L. Delgado-Baquerizo M. Wang J. Zhu Y. Wang R.. (2022). Fungi drive soil multifunctionality in the coastal salt marsh ecosystem. Sci. Total Environ. 818:151673. 10.1016/j.scitotenv.2021.151673 34793796

[B35] Li J. Sang C. Yang J. Qu L. Xia Z. Sun H.. (2021). Stoichiometric imbalance and microbial community regulate microbial elements use efficiencies under nitrogen addition. Soil Biol. Biochem. 156:108207. 10.1016/j.soilbio.2021.108207

[B36] Li J. Wei G. Wang N. Gao Z. (2014). Diversity and distribution of nirK-harboring denitrifying bacteria in the water column in the Yellow River estuary. Microb. Environ. 29, 107–110. 10.1264/jsme2.ME13111 24621509 PMC4041238

[B37] Li X. Qian W. Hou L. Liu M. Chen Z. Tong C.. (2020a). Soil organic carbon controls dissimilatory nitrate reduction to ammonium along a freshwater-oligohaline gradient of Min River Estuary, Southeast China. Marine Pollut. Bull. 160:111696. 10.1016/j.marpolbul.2020.111696 33181963

[B38] Li X. Sun Z. Tian L. He T. Li J. Wang J.. (2020b). Effects of spatial expansion between phragmites australis and cyperus malaccensis on variations of arsenic and heavy metals in decomposing litters in a typical subtropical estuary (Min River), China. Chemosphere 240:124965. 10.1016/j.chemosphere.2019.124965 31726610

[B39] Li Y. Li W. Jiang L. Li E. Yang X. Yang J.. (2024). Salinity affects microbial function genes related to nutrient cycling in arid regions. Front. Microbiol. 15:1407760. 10.3389/fmicb.2024.1407760 38946896 PMC11212614

[B40] Liang H. Huang J. Xia Y. Yang Y. Yu Y. Zhou K.. (2024). Spatial distribution and assembly processes of bacterial communities in riverine and coastal ecosystems of a rapidly urbanizing megacity in China. Sci. Total Environ. 934:173298. 10.1016/j.scitotenv.2024.173298 38761945

[B41] Lin Y. Hu H. W. Deng M. Yang P. Ye G. (2023). Microorganisms carrying I and II share similar ecological niches in a subtropical coastal wetland. Sci. Total Environ. 870:162008. 10.1016/j.scitotenv.2023.162008 36739025

[B42] Liu J. Cade-Menun B. J. Yang J. Hu Y. Liu C. W. Tremblay J.. (2018). Long-term land use affects phosphorus speciation and the composition of phosphorus cycling genes in agricultural soils. Front. Microbiol. 9:1643. 10.3389/fmicb.2018.01643 30083148 PMC6065304

[B43] Liu J. Guo Y. Gu H. Liu Z. Hu X. Yu Z.. (2023). Conversion of steppe to cropland increases spatial heterogeneity of soil functional genes. ISME J. 17, 1872–1883. 10.1038/s41396-023-01496-9 37607984 PMC10579271

[B44] Liu Z. Jin S. Yu H. Wang W. Shen J. He J.. (2022). Effect of land use change on the eco-stoichiometric characteristics in Min River estuary wetland. J. Soil Water Conserv. 36, 410–416. 10.13870/j.cnki.stbcxb.2022.06.050

[B45] Lorrain-Soligon L. Robin F. Bertin X. Jankovic M. Rousseau P. Lelong V.. (2023). Long-term trends of salinity in coastal wetlands: effects of climate, extreme weather events, and sea water level. Environ. Res. 237:116937. 10.1016/j.envres.2023.116937 37611783

[B46] Luo M. Zeng C-. S. Tong C. Huang J-. F. Chen K. Liu F-. Q.. (2016). Iron reduction along an inundation gradient in a tidal sedge (cyperus malaccensis) marsh: the rates, pathways, and contributions to anaerobic organic matter mineralization. ESCO 39, 1679–1693. 10.1007/s12237-016-0094-0

[B47] Luo S. Yuan J. Song Y. Ren J. Qi J. Zhu M.. (2025). Elevated salinity decreases microbial communities complexity and carbon, nitrogen and phosphorus metabolism in the Songnen Plain wetlands of China. Water Res. 276:123285. 10.1016/j.watres.2025.123285 39954460

[B48] Manoharan L. Kushwaha S. K. Ahrén D. Hedlund K. (2017). Agricultural land use determines functional genetic diversity of soil microbial communities. Soil Biol. Biochem. 115, 423–432. 10.1016/j.soilbio.2017.09.011

[B49] Mao D. Luo L. Wang Z. Wilson M. C. Zeng Y. Wu B.. (2018). Conversions between natural wetlands and farmland in China: A multiscale geospatial analysis. Sci. Total Environ. 634, 550–560. 10.1016/j.scitotenv.2018.04.009 29635197

[B50] Mohapatra M. Yadav R. Rajput V. Dharne M. S. Rastogi G. (2021). Metagenomic analysis reveals genetic insights on biogeochemical cycling, xenobiotic degradation, and stress resistance in mudflat microbiome. Journal of Environmental Management 292:112738. 10.1016/j.jenvman.2021.112738 34020306

[B51] Mutlu M. B. Martíez-García M. Santos F. Peña A. Guven K. Antón J.. (2008). Prokaryotic diversity in Tuz Lake, a hypersaline environment in Inland Turkey. FEMS Microbiol. Ecol. 65, 474–483. 10.1111/j.1574-6941.2008.00510.x 18537839

[B52] O'Brien F. J. M. Almaraz M. Foster M. A. Hill A. F. Huber D. P. King E. K.. (2019). Soil salinity and pH drive soil bacterial community composition and diversity along a lateritic slope in the avon river critical zone observatory, Western Australia. Front. Microbiol. 10:1486. 10.3389/fmicb.2019.01486 31312189 PMC6614384

[B53] Pelikan C. Herbold C. W. Hausmann B. Müller A. L. Pester M. Loy A.. (2016). Diversity analysis of sulfite- and sulfate-reducing microorganisms by multiplex dsrA and dsrB amplicon sequencing using new primers and mock community-optimized bioinformatics. Environ. Microbiol. 18, 2994–3009. 10.1111/1462-2920.13139 26625892

[B54] Peng Z. Qian X. Liu Y. Li X. Gao H. An Y.. (2024). Land conversion to agriculture induces taxonomic homogenization of soil microbial communities globally. Nat. Commun. 15:3624. 10.1038/s41467-024-47348-8 38684659 PMC11058813

[B55] Schimel J. Balser T. C. Wallenstein M. (2007). Microbial stress-response physiology and its implications for ecosystem function. Ecology 88, 1386–1394. 10.1890/06-0219 17601131

[B56] Shen J. Yu D. Liu Z. Di H. He J-. Z. (2024). Land use conversion to uplands significantly increased the risk of antibiotic resistance genes in estuary area. Environ. Int. 191:108953. 10.1016/j.envint.2024.108953 39153385

[B57] Sheng Q. Wang L. Wu J. (2015). Vegetation alters the effects of salinity on greenhouse gas emissions and carbon sequestration in a newly created wetland. Ecol. Eng. 84, 542–550. 10.1016/j.ecoleng.2015.09.047

[B58] Siles J. A. Margesin R. (2016). Abundance and diversity of bacterial, archaeal, and fungal communities along an altitudinal gradient in alpine forest soils: what are the driving factors? Microb. Ecol. 72, 207–220. 10.1007/s00248-016-0748-2 26961712 PMC4902835

[B59] Smith C. J. Nedwell D. B. Dong L. F. Osborn A. M. (2007). Diversity and abundance of nitrate reductase genes (narG and napA), nitrite reductase genes (nirS and nrfA), and their transcripts in estuarine sediments. Appl. Environ. Microbiol. 73, 3612–3622. 10.1128/AEM.02894-06 17400770 PMC1932689

[B60] Smith P. Cotrufo M. F. Rumpel C. Paustian K. Kuikman P. J. Elliott J. A.. (2015). Biogeochemical cycles and biodiversity as key drivers of ecosystem services provided by soils. Soil 1, 665–685. 10.5194/soil-1-665-201529600493

[B61] Sun Y. Li X. Mander Ü. He Y. Jia Y. Ma Z.. (2011). Effect of reclamation time and land use on soil properties in changjiang river estuary, China. Chin. Geogra. Sci. 21, 403–416. 10.1007/s11769-011-0482-0

[B62] Sun Z. Sun W. Tong C. Zeng C. Yu X. Mou X.. (2015). China's coastal wetlands: conservation history, implementation efforts, existing issues and strategies for future improvement. Environ. Int. 79, 25–41. 10.1016/j.envint.2015.02.017 25771079

[B63] Tan L. Ge Z. Ji Y. Lai D. Y. F. Temmerman S. Li S.. (2022). Land use and land cover changes in coastal and inland wetlands cause soil carbon and nitrogen loss. Glob. Ecol. Biogeogr. 31, 2541–2563. 10.1111/geb.13597

[B64] Tang C. Liu Y. Li Z. Guo L. Xu A. Zhao J.. (2021). Effectiveness of vegetation cover pattern on regulating soil erosion and runoff generation in red soil environment, southern China. Ecol. Indic. 129:107956. 10.1016/j.ecolind.2021.107956

[B65] Temmink R. J. M. Lamers L. P. M. Angelini C. Bouma T. J. Fritz C. van de Koppel J.. (2022). Recovering wetland biogeomorphic feedbacks to restore the world's biotic carbon hotspots. Science 376:eabn1479. 10.1126/science.abn1479 35511964

[B66] Tian D. Xiang Y. Seabloom E. Chen H. Y. H. Wang J. Yu G.. (2022). Ecosystem restoration and belowground multifunctionality: a network view. Ecol. Appl. 32:e2575. 10.1002/eap.2575 35191122

[B67] Tong C. Wang W. Q. Zeng C. S. Marrs R. (2010). Methane (CH4) emission from a tidal marsh in the Min River estuary, southeast China. J. Environ. Sci. Health 45, 506–516. 10.1080/10934520903542261 20390897

[B68] Wagg C. Bender S. F. Widmer F. van der Heijden M. G. A. (2014). Soil biodiversity and soil community composition determine ecosystem multifunctionality. PNAS 111, 5266–5270. 10.1073/pnas.1320054111 24639507 PMC3986181

[B69] Wang K. Wang C. Feng X. M. Wu X. Fu B. J. (2022). Research progress on the relationship between biodiversity and ecosystem multifunctionality. Acta Ecol. Sin. 42, 11–23. 10.5846/stxb202105141263

[B70] Wang Z. Song S. Song T. Yuan L. Zhang C. (2022). Responses of edaphic factors and microbial community to terrestrial succession and experimental warming in coastal salt marshes. Pedobiologia 93–94:150821. 10.1016/j.pedobi.2022.150821

[B71] Wu B. Ding M. Zhang H. Devlin A. T. Wang P. Chen L.. (2023). Reduced soil multifunctionality and microbial network complexity in degraded and revegetated alpine meadows. J. Environ. Manage. 343:118182. 10.1016/j.jenvman.2023.118182 37224687

[B72] Wu T. Milner H. Díaz-Pérez J. C. Ji P. (2015). Effects of soil management practices on soil microbial communities and development of southern blight in vegetable production. Appl. Soil Ecol. 91, 58–67. 10.1016/j.apsoil.2015.02.011

[B73] Xu X. Liu Y. Singh B. P. Yang Q. Zhang Q. Wang H.. (2020). NosZ clade II rather than clade I determine in situ N2O emissions with different fertilizer types under simulated climate change and its legacy. Soil Biol. Biochem. 150:107974. 10.1016/j.soilbio.2020.107974

[B74] Yao R. Yang J. Wang X. Xie W. Zheng F. Li H.. (2021). Response of soil characteristics and bacterial communities to nitrogen fertilization gradients in a coastal salt-affected agroecosystem. Land Degrad. Dev. 32, 338–353. 10.1002/ldr.3705

[B75] Zeng C. Zhong C. Tong C. Liu Z. (2008). Impacts of LUCC on soil organic carbon contents in wetland of Minjiang river estuary. J. Soil Water Conserv. 22, 125–129. 10.13870/j.cnki.stbcxb.2008.05.030

[B76] Zhang G. Bai J. Tebbe C. C. Zhao Q. Jia J. Wang W.. (2020). Salinity controls soil microbial community structure and function in coastal estuarine wetlands. Environ. Microbiol. 23, 1020–1037. 10.1111/1462-2920.15281 33073448

[B77] Zhang G. Bai J. Zhai Y. Jia J. Zhao Q. Wang W.. (2024). Microbial diversity and functions in saline soils: a review from a biogeochemical perspective. J. Adv. Res. 59, 129–140. 10.1016/j.jare.2023.06.015 37392974 PMC11081963

[B78] Zhang W-. L. Zeng C-. S. Tong C. Zhai S-. J. Lin X. Gao D-. Z.. (2015). Spatial distribution of phosphorus speciation in marsh sediments along a hydrologic gradient in a subtropical estuarine wetland, China. Estuar. Coast. Shelf Sci. 154, 30–38. 10.1016/j.ecss.2014.12.023

[B79] Zhang X. Wang L. Zhou W. Hu W. Hu J. Hu M.. (2022). Changes in litter traits induced by vegetation restoration accelerate litter decomposition in plantations. Land Degrad. Dev. 33, 179–192. 10.1002/ldr.4136

[B80] Zheng B. Zhu Y. Sardans J. Peñuelas J. Su J. (2018). QMEC: a tool for high-throughput quantitative assessment of microbial functional potential in C, N, P, and S biogeochemical cycling. Sci. China Life Sci. 61, 1451–1462. 10.1007/s11427-018-9364-7 30136056

[B81] Zhu B. Whalen J. K. Wu J. Yang J. Mao X. Wan B.. (2024). Soil food web structure coordinated by soil omnivores sustains soil multifunctionality in moderate vermicompost amended fields. Soil Biol. Biochem. 192:109391. 10.1016/j.soilbio.2024.109391

[B82] Zhu X. Yuan F. He L. Guo Z. Wang N. Zuo Y.. (2022). Wetland conversion to cropland alters the microbes along soil profiles and over seasons. Catena 214:106282. 10.1016/j.catena.2022.106282

[B83] Zhu Y. Song X. Liu X. Chen W. Niu X. Zhou W.. (2022). Influences of land reclamation on soil bacterial communities of abandoned salt pans in the Yellow River Delta. Land Degrad. Dev. 33, 3231–3244. 10.1002/ldr.4384

