# Supplementary material for: Land use changes alter microbial functional gene diversity and its relationship with soil ecosystem multifunctionality in a subtropical estuary
Source: Front Microbiol. 2025 Jun 3;16:1592901. doi: 10.3389/fmicb.2025.1592901 (PMC12170521; doi:10.3389/fmicb.2025.1592901)

**Supplementary materials**

**Land use changes alter microbial functional gene diversity and its relationship with soil ecosystem multifunctionality in a subtropical estuary**

Zi-Kai Liu ^a, #^, Lige Zhang ^a, #^, Shengsheng Jin ^a^, Hanxia Yu ^a^, Ji-Zheng He ^a,b^, Ju-Pei Shen ^a,b*^

^a^ Key Laboratory of Humid Subtropical Eco-geographical Process of Ministry of Education, Fujian Normal University, Fuzhou 350117, China

^b^ School of Geographical Sciences/School of Carbon Neutrality Future Technology, Fujian Normal University, Fuzhou 350117, China

*Corresponding author: Ju-Pei Shen

School of Geographical Sciences, School of Carbon Neutrality Future Technology, Fujian Normal University, Fuzhou 350117 China

Email addresses: [jpshen@fjnu.edu.cn](mailto:jpshen@fjnu.edu.cn) , 86 15910236130 (tel)

# These authors contributed equally to this work.

Type of contribution: Research article

**Table S1**

Soil properties, microbial biomass and enzyme activities across different land uses

| Land use | RW | TF | GR | AL | FL |
| --- | --- | --- | --- | --- | --- |
| EC (mS·cm^-1^） | 4.53 (0.41) | 5.73 (0.32) | 1.99 (0.32) | 0.31 (0.19) | 0.29 (0.04) |
| NH_4_^+^-N (mg·kg^-1^） | 4.11 (0.69) | 3.69 (0.08) | 2.49 (0.33) | 14.45 (2.42) | 4.74 (0.35) |
| NO_3_^-^-N (mg·kg^-1^） | 1.74 (0.27) | 1.83 (0.35) | 0.67 (0.05) | 6.84 (1.13) | 1.51 (0.47) |
| AP (mg·kg^-1^） | 6.72 (0.71) | 5.89 (0.76) | 5.42 (1.46) | 5.67 (1.61) | 6.25 (1.81) |
| pH (H_2_O) | 6.22 (0.03) | 6.33 (0.09) | 5.47 (0.01) | 7.52 (0.18) | 6.45 (0.15) |
| TP (g·kg^-1^） | 0.59 (0.01) | 0.51 (0.02) | 0.38 (0.04) | 0.32 (0.01) | 0.55 (0.03) |
| TC (g·kg^-1^） | 26.10 (0.25) | 15.92 (0.65) | 11.92 (1.62) | 14.78 (1.11) | 38.72 (0.87） |
| TN (g·kg^-1^） | 2.09 (0.03） | 1.30 (0.03) | 1.15 (0.21) | 1.37 (0.12) | 3.05 (0.09) |
| DON (mg·kg^-1^） | 41.29 (1.16) | 13.22 (0.81) | 6.39 (0.43) | 31.63 (3.16) | 35.66 (6.92) |
| DOC (mg·kg^-1^） | 60.78 (3.08) | 35.56 (3.24) | 27.85 (5.65) | 74.18 (4.10) | 73.89 (7.71) |
| MBC (mg·kg^-1^） | 326.02 (22.79) | 131.29 (27.43) | 149.51 (17.34) | 232.94 (18.02) | 1272.67 (275.53) |
| MBN (mg·kg^-1^） | 42.58 (6.64) | 20.43 (1.99) | 20.61 (4.56) | 22.63 (3.84) | 124.36 (19.00) |
| ACP μmol·(g^-1^·h^-1^) | 1529.94 (636.92) | 210.43  (22.49) | 245.22  (45.40) | 1229.49  (619.24) | 3856.44  (589.73) |
| βG μmol·(g^-1^·h^-1^) | 493.25 (65.99) | 78.44 (4.21) | 32.32 (3.19) | 126.95 (50.60) | 385.11 (64.28) |
| NAG μmol·(g^-1^·h^-1^) | 403.43 (37.39) | 70.36 (4.83) | 24.46 (2.11) | 185.22 (67.85) | 433.29 (113.13) |
| LAP μmol·(g^-1^·h^-1^) | 31.97 (5.62) | 10.31 (2.44) | 26.35 (16.65) | 34.43 (14.09) | 175.61 (35.29) |

*Note*: Data as mean (sd) (n = 3), EC: electrical conductivity, NH_4_^+^-N: ammonium nitrogen, NO_3_^-^-N: nitrate nitrogen, AP: Available phosphorus, DON: dissolved organic nitrogen, DOC: dissolved organic carbon, MBC: microbial biomass carbon, MBN: microbial biomass nitrogen, ACP: acid phosphatase, βG: β-1,4-glucosidase, NAG: β-1,4-N-acetylglucosaminidase, LAP: L-leucine aminopeptidase, pH: power of hydrogen, TP: total phosphorus, TC: total carbon, TN: total nitrogen. RW: reed wetland; TF: tidal flat; GR: grassland; AL: agricultural land; FL: fallow land.

**Table S2**

Table S2 The primer pair information for each gene

| Gene name | Forward primer | Reverse primer | Gene name | Forward primer | Reverse primer |
| --- | --- | --- | --- | --- | --- |
| *16S rRNA* | GGGTTGCGCTCGTTGC | ATGGYTGTCGTCAGCTCGTG | *nirS1* | GTSAACGTSAAGGARACSGG | GASTTCGGRTGSGTCTTGA |
| *rbcL* | AAGGACGACGAGAACATC | TGCAGGATCATGTCGTT | *nirS2* | ATCGTCAACGTCAARGARACVGG | TTCGGGTGCGTCTTSABGAASAG |
| *aclB* | TGGACMATGGTDGCYGGKGGT | ATAGTTKGGSCCACCTCTTC | *nirS3* | TGGAGAACGCCGGNCARGTNTGG | GATGATGTCCACGGCNACRTANGG |
| *korA* | GCCGGCTACCCCATCACCCC | ATGATGGGATGGTCGCCATG | *nirK1* | GGMATGGTKCCSTGGCA | GCCTCGATCAGRTTRTGGTT |
| *acsA* | GATACCTGGTGGCAGACCGA | TGATCACGTCGTCGACCCGG | *nirK2* | ATGGCGCCATCATGGTNYTNCC | TCGAAGGCCTCGATNARRTTRTG |
| *acsE* | TCATCGGCGAACGCATCAAC | AGRCCGGCTTCSATGGC | *nirK3* | TGCACATCGCCAACGGNATGTWYGG | GGCGCGGAAGATGSHRTGRTCNAC |
| *acsB* | CTYTGYCAGTCMTTYGCBCC | CCCATAAABCCYGGDGTYTG | *nosZ2* | CGYTGTTCMTCGACAGCCAG | CGSACCTTSTTGCCSTYGCG |
| *accA* | GAAGGCTAYCGCAARGC | CCTTCMGGSGARATMAC | *nosZ1* | CGCRACGGCAASAAGGTSMSSGT | CAKRTGCAKSGCRTGGCAGAA |
| *pccA* | GTGMTGATCAAGGCCWC | CGSGTGTTCATYTCSAGGAA | *ppk* | GACCCGAABGTRCTBGCSAT | TTATAATTNCCSGTNCCNA |
| *smtA* | TTTCTGGCCGGBTAYGCDGC | CGGTACGGHCCGGTYTGVCC | *ppx* | TGCATCTGGCGGACGGCCT | AGATCCGCCGCCAATATCA |
| *mct* | TGGGCGCSGASGTSATMCG | TTGACSGTRTARTCSAYSGC | *phoD* | CAGTGGGACGACCACGAGGT | GAGGCCGATCGGCATGTCG |
| *frdA* | MTGCTGCACACSCTGTW | CCGGTSGGGTGRWACTG | *phoX* | GARGAGAACWTCCACGGYTA | GATCTCGATGATRTGRCCRAAG |
| *mcrA* | GGTGGTGTMGGDTTCACMCARTA | CGTTCATBGCGTAGTTVGGRTAGT | *bpp* | GACGCAGCCGAYGAYCCNGCNITNTGG | CAGGSCGCANRTCIACRTTRTT |
| *mmoX* | ATGGAGGCGGTCAAGGACGA | CGCTTCATGCCCTTCCACAG | *cphy* | GTGGACCTRCGRMARGARWCICA | GTCCGACCATTGCCTGCYTCRCAR  TGRAMRTGIADCCA |
| *pmoA* | GGNGACTGGGACTTCTGG | GAASGCNGAGAAGAASGC | *pqqC* | AACCGCTTCTACTACCAG | GCGAACAGCTCGGTCAG |
| *mxaF* | GCGGCACCAACTGGGGCTGGT | GGGCAGCATGAAGGGCTCCC | *gcd* | ATCGCGTTCGGGCCGGACG | ATSAGRTTSAGCTCGTCCCA |
| *sga* | CGSAACTGGGAYTACCGS | TCCCACAGSCCSKCGTC | *pqq-mdh* | TGTTCTATGTGCCGGCCAA | CTTCCACAGTTCCTTGCC |
| *abfA* | CGSTAYCCSGGCGGCAAYTT | TGCCASGGNCCGTCCATYTC | *phnK* | CATCGTCGGCGAATCCGG | TGCTGCATGCCGCCGGAAAA |
| *xylA* | TGGGGBGGTCGYGAAGG | ACTTTGGCRTCRAAGTT | *dsrA* | ACSCACTGGAAGCACG | gGTGGAGCCGTGCATGTT |
| *exg* | YSTACGGSATGCACTGGMT | TANCGCAGRTAGTCVCCCAT | *dsrB* | CAACATCGTYCAYACCCAGGG | GTGTAGCAGTTACCGCA |
| *pgu* | ANCATTGGTGGCCSTGGAA | TTRAYGGCRATRCARTCRTC | *aspA* | GGGYCTKTCCGCYATCAAYAC | ATCATGATCTGCCAgCGgCCGGA |
| *mnp* | MACRCCSTTCGACTCSACC | ACGTCSGAGCAGTCRAYGA | *yedZ* | CTGCTGATCACGCTGGCCAT | GCGATGCAGCTTCTTCCAGCG |
| *ghdA* | GCCATCGGYCCWTACAAGGG | ATGTCRCCNGCCGGAACGTC | *soxY* | ATCGATGACAACCCCGTGCC | AGCTGGTCCATCTGCATGCCG |
| *UreC* | AAGMTSCACGAGGACTGGGG | AGRTGGTGGCASACCATSAGCAT | *iso-plu* | GTCATYTACTTYGGNCC | CGNGCSACATCNGCCCA |
| *nifH* | AAAGGYGGWATCGGYAARTCCACCAC | TGSGCYTTGTCYTCRCGGATBGGCAT | *amyX* | TATAAYTGGGGMTATGAYCC | CCCATYAAATCAAAWCGRAA |
| *hzo* | AAGACNTGYCAYTGGGGWAAA | GACATACCCATACTKGTRTANACNGT | *apu* | ACVTGGATAGGYGAGCCYCA | CCRTCSGGGAAGTAGTTKCC |
| *hzsA* | WTYGGKTATCARTATGTAG | AAABGGYGAATCATARTGGC | *cdaR* | CGARATGGTGGTGCTCAA | CARCGTRTTACGATGAATA |
| *hzsB* | ARGGHTGGGGHAGYTGGAAG | GTYCCHACRTCATGVGTCTG | *amyA* | YGGTTTTCGTCTTGACGCSG | MGGCTGMGTRTCATGRTTK |
| *amoA1* | STAATGGTCTGGCTTAGACG | GCGGCCATCCATCTGTATGT | *manB* | ATGCGCGGBGTCAACCA | TCGTTGSCGATGTTGABGA |
| *amoA2* | GGGGTTTCTACTGGTGGT | CCCCTCKGSAAAGCCTTCTT | *cdh* | ATWRYCTWCCGMRTHGCCMT | GTKAGSGGRTTBYKGRYCAT |
| *amoB* | TGGTAYGACATKAWATGG | RCGSGGCARGAACATSGG | *naglu* | TVAAYTGGTAYCTGAAATAY | CCRTGYAGVGCCATCCAGTC |
| *Hao* | TGTCATACCCGGCACAAGTTC | CATRTGGCAGAACTGRCABGT | *chiA* | TSAAGAARTACGCSGACAACG | ASGTCATCAGRCCCTTSAG |
| *nxrA* | CAGACCGACGTGTGCGAAAG | TCCACAAGGAACGGAAGGTC | *exo-chi* | GATTGGTSVCAATATGAYRG | STCCARCCACCRAYRCTRAA |
| *narG* | TAYGTSGGGCAGGARAAACTG | CGTAGAAGAAGCTGGTGCTGT | *glx* | AACCAGTCGATCATCTACGA | RTGSACGAGCTCDGGCATGG |
| *napA* | CTGGACIATGGGYTTIAACCA | CCTTCYTTYTCIACCCACAT | *lig* | CCGCACACACTGTTGCTGC | CGAAGGATTGCCACTCGCA |
| *nasA* | CARCCNAAYGCNATGGG | ATNGTRTGCCAYTGRTC | *pox* | ACYAGTATCCATTGGCACGGT | AGATGVGARTGATACCARAA |

*Note:* Primers information cited from Zheng et al. (2018).

Zheng, B., Zhu, Y., Sardans, J., Peñuelas, J., and Su, J. (2018). QMEC: a tool for high-throughput quantitative assessment of microbial functional potential in C, N, P, and S biogeochemical cycling. *Science China Life Sciences* 61(12)**,** 1451-1462. doi: 10.1007/s11427-018-9364-7.

**Fig. S1** Percentage of relative abundance of top microbial 10 genes in all the detected genes (a) and chord diagram analysis at top 10 genes (d) based on their abundance in different land use. RW: reed wetland; TF: tidal flat; GR: grassland; AL: agricultural land; FL: fallow land. Different colors represent different variables.


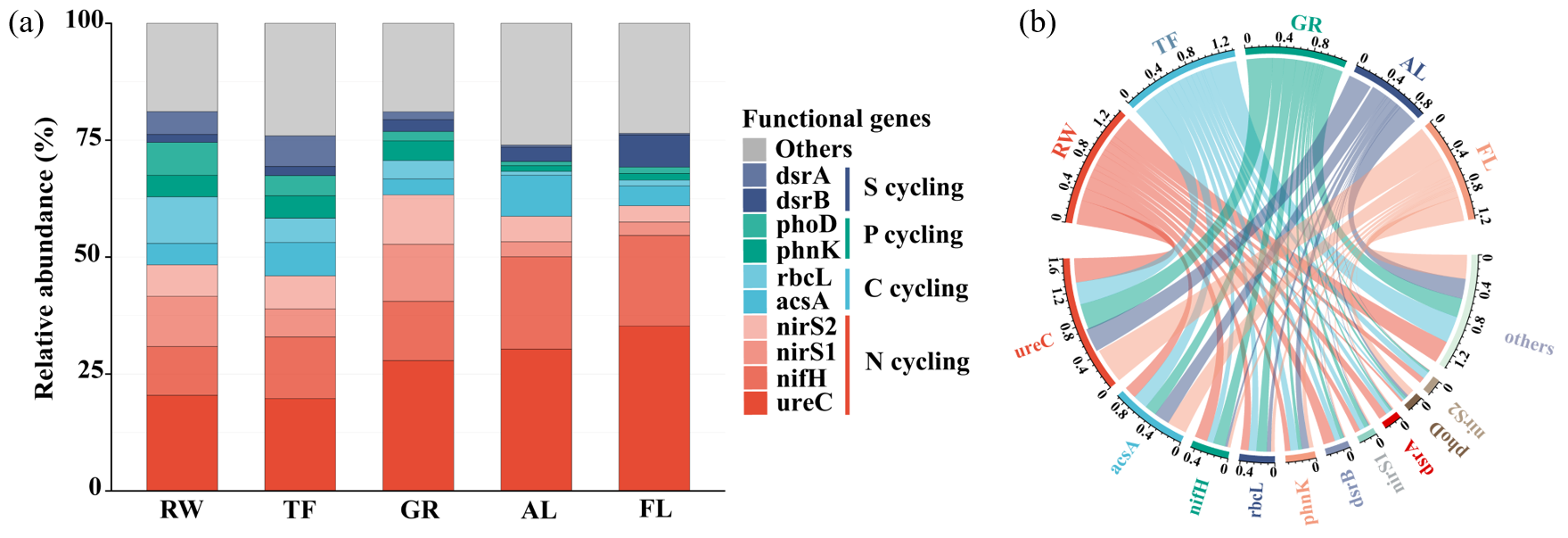


**Fig. S2** Regression analysis of EC, microbial functional gene richness and NMDS1. The shady region shows the regression model's 95 % confidence interval. Different colors represent different variables.


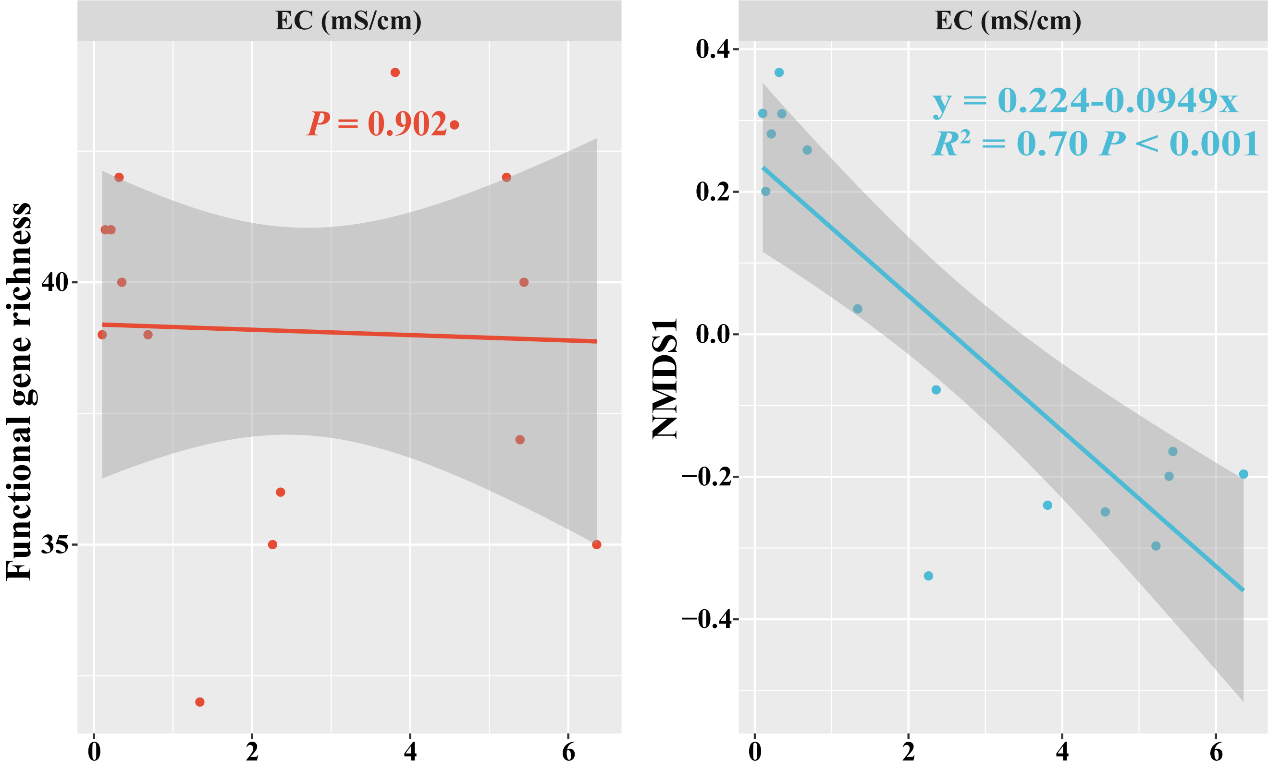


**Fig. S3**

Soil EMF in different land uses. Different letters indicate significant differences among different land use (*P* < 0.05). RW: reed wetland; TF: tidal flat; GR: grassland; AL: agricultural land; FL: fallow land.


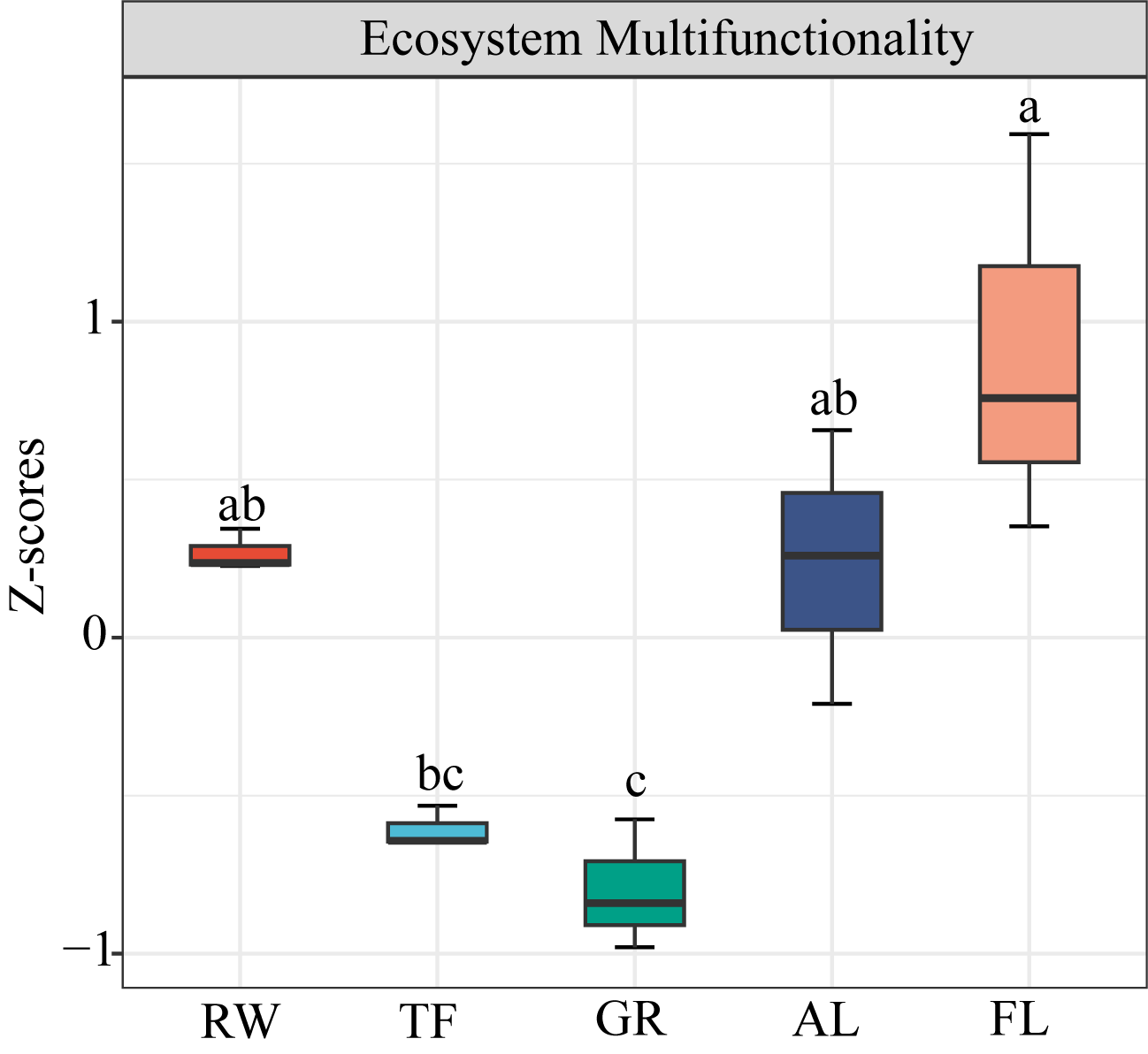


**Fig. S4** Random forest analysis of top 10 functional genes affecting EMF. Significance levels are denoted with **P* < 0.05, ***P* < 0.01and ****P* < 0.001. Different colors represent different genes.


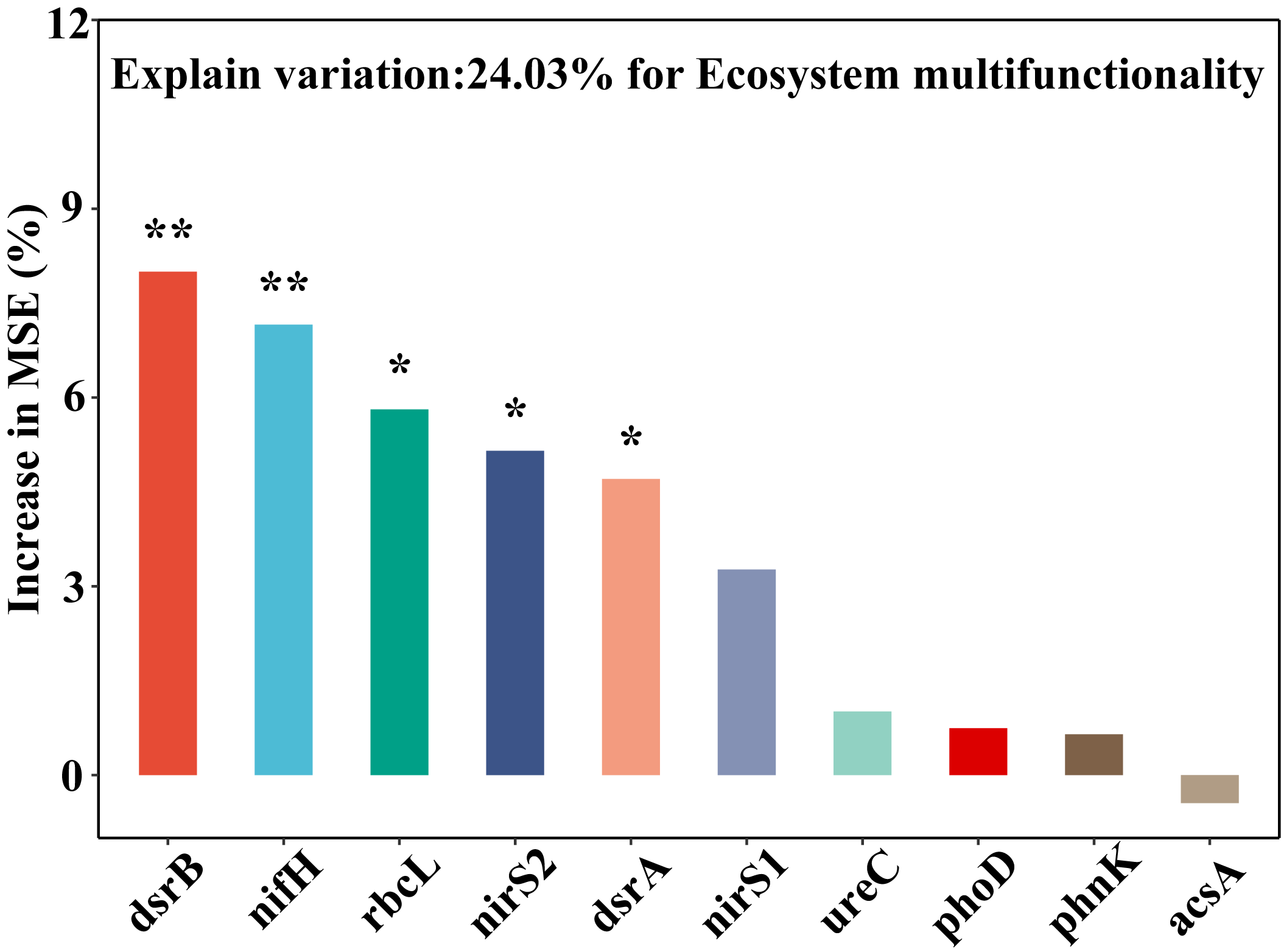

Supplement: Supplementary file 1 [file Data_Sheet_1.docx]
